# Combination of Berberine with Resveratrol Improves the Lipid-Lowering Efficacy

**DOI:** 10.3390/ijms19123903

**Published:** 2018-12-06

**Authors:** Xiaofei Zhu, Jingyi Yang, Wenjuan Zhu, Xiaoxiao Yin, Beibei Yang, Yihui Wei, Xiaofang Guo

**Affiliations:** 1 Department of Clinical immunology, School of Laboratory Medicine, Xinxiang Medical University, Xinxiang 453003, China; yjy289688354@163.com (J.Y.); zzwwjj0304@163.com (W.Z.); xiao143134@163.com (X.Y.); ybbcyh@163.com (B.Y.); weiyihui1@163.com (Y.W.); 2 Henan Collaborative Innovation Center of Molecular Diagnosis and Laboratory Medicine, Xinxiang Medical University, Xinxiang 453003, China; 3 Henan Key Laboratory of Immunology and Targeted Drugs, Xinxiang Medical University, Xinxiang 453003, China; 4 Department of Microbiology, School of Basic Medical Sciences, Xinxiang Medical University, Xinxiang 453003, China

**Keywords:** berberine, resveratrol, sirtuin 1, high fat diet, low-density-lipoprotein receptor

## Abstract

The natural compound berberine has been reported to exhibit anti-diabetic activity and to improve disordered lipid metabolism. In our previous study, we found that such compounds upregulate expression of sirtuin 1—a key molecule in caloric restriction, it is, therefore, of great interest to examine the lipid-lowering activity of berberine in combination with a sirtuin 1 activator resveratrol. Our results showed that combination of berberine with resveratrol had enhanced hypolipidemic effects in high fat diet-induced mice and was able to decrease the lipid accumulation in adipocytes to a level significantly lower than that in monotherapies. In the high fat diet-induced hyperlipidemic mice, combination of berberine (30 mg/kg/day, oral) with resveratrol (20 mg/kg/day, oral) reduced serum total cholesterol by 27.4% ± 2.2%, and low-density lipoprotein-cholesterol by 31.6% ± 3.2%, which was more effective than that of the resveratrol (8.4% ± 2.3%, 6.6% ± 2.1%) or berberine (10.5% ± 1.95%, 9.8% ± 2.58%) monotherapy (*p* < 0.05 for both). In 3T3-L1 adipocytes, the treatment of 12 µmol/L or 20 µmol/L berberine combined with 25 µmol/L resveratrol showed a more significant inhibition of lipid accumulation observed by Oil red O stain compared with individual compounds. Moreover, resveratrol could increase the amount of intracellular berberine in hepatic L02 cells. In addition, the combination of berberine with resveratrol significantly increases the low-density-lipoprotein receptor expression in HepG2 cells to a level about one-fold higher in comparison to individual compound. These results implied that the enhanced effect of the combination of berberine with resveratrol on lipid-lowering may be associated with upregulation of low-density-lipoprotein receptor, and could be an effective therapy for hyperlipidemia in some obese-associated disease, such as type II diabetes and metabolic syndrome.

## 1. Introduction

Obesity is recognized as a major risk factor for clinically obesity-associated cardiometabolic complications, including metabolic syndrome components, type 2 diabetes, and cardiovascular diseases [1,2]. Excessive adiposity, especially visceral fat, as an endocrine organ, is associated with overmuch plasma free fatty acid-induced lipotoxicity and a chronic proinflammatory state by releasing excessive inflammatory cytokines, such as tumor necrosis factor-α (TNF-α), resulting in insulin resistance, hyperglycemia, dyslipidemia, hypertension, and a prothrombotic state [3,4,5]. Therefore, ameliorating dyslipidemia and fat deposition may contribute to reverse the chronic proinflammatory state, and then to delay or prevent the morbidity of these obese-associated diseases.

Berberine (Ber), an active nature component of traditional Chinese herb *Coptidis Rhizoma*, attracted many researchers’ attention as a new hypolipidemic drug, which showed potent effect against hyper-cholesteremia and type II diabetes. Moreover, Ber reduced adipocytes differentiation and lipid accumulation by inhibiting the expression of lipogenic genes. However, transient gastrointestinal adverse effects, including diarrhea, flatulence, constipation, could be observed in clinical trials because of a high dose of Ber alone (500 mg/2 or 3 times a day) [5,6,7,8]. For example, 10.3% diarrhea, 19.0% flatulence, and 6.9% constipation were occurred in patients after administration of Ber alone in a clinical trial. In addition, all of these adverse effects disappeared in one week after reduction in berberine dosage [9]. In our previous study, it was found that Ber could resist oxidative stress induced apoptosis or cellular senescence by upregulation of sirtuin 1 (SIRT1) expression at a low dosage [10,11]. SIRT1 was regarded as a master metabolic sensor by mimicking the beneficial effect of caloric restriction in mammals [12]. Resveratrol (Res), a chemical activator of SIRT1, also exhibited improvement of lipid metabolism in pathologic state, such as obesity, type II diabetes and atherosclerosis [13,14]. Therefore, in this study, we investigated the effect of Ber in combination with Res on lipid metabolism in hyperlipidemia animal and 3T3-L1 adipocytes. The possible enhanced mechanism of Ber in combination with Res was also studied.

## 2. Results

### 2.1. The Anti-Hyperlipidemic Effect of Combined Therapy with Berberine and Resveratrol in Hyperlipidemic Mice and 3T3-L1 Adipocytes

**Figure 1:**
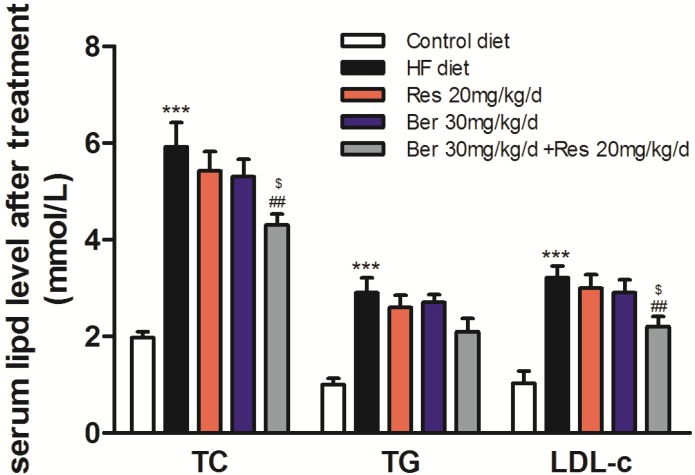
The lipid-lowering effect of Ber, Res and their combination in hyperlipidemic C57BL/6J mice. As Materials and methods mentioned, serums were isolated from mice after different drug treatments. The total cholesterol (TC), Triglyceride (TG) and LDL-cholesterol (LDL-c) levels were measured. Values are mean ± SD of all of mice in each group (*n* = 8). *** *p* < 0.001 vs. that of the control low diet group; ^##^ *p* < 0.01 vs. that of the HFD group; ^$^ *p* < 0.05 vs. that of Ber or Res monotherapy. The result is a representative for three separate experiments.

The level of plasma total cholesterol (TC), triglyceride (TG) and low-density lipoprotein-cholesterol (LDL-c) in high fat diet (HFD) mice after administration of Ber and/or Res was shown in Figure 1. Compared with control low fat diet (LFD) group, the level of plasma TC, TG and LDL-c in HFD group were significantly increased (*p* < 0.01). Compared with HFD group administrated with equal volume of 0.9% saline, the level of TC, TG and LDL-c were decrease by 27.4% ± 2.2%, 17.2% ± 2.42% and 31.6% ± 3.2%, respectively after administration of 30 mg/kg/day Ber in combination with 20 mg/kg/day Res. However, the monotherapy of Res or Ber showed an 8.4% ± 2.3% or 10.5% ± 1.95% reduction in TC, 10.3% ± 3.42% or 6.9% ± 2.6% reduction in TG and 6.6% ± 2.1% or 9.8% ± 2.58% reduction in LDL-c, respectively. The combination therapy demonstrated a superior hypolipidemic efficacy in level of plasma TC and LDL-c to monotherapy (*p* < 0.05), but there was no differentiation in plasma TG. 

**Figure 2:**
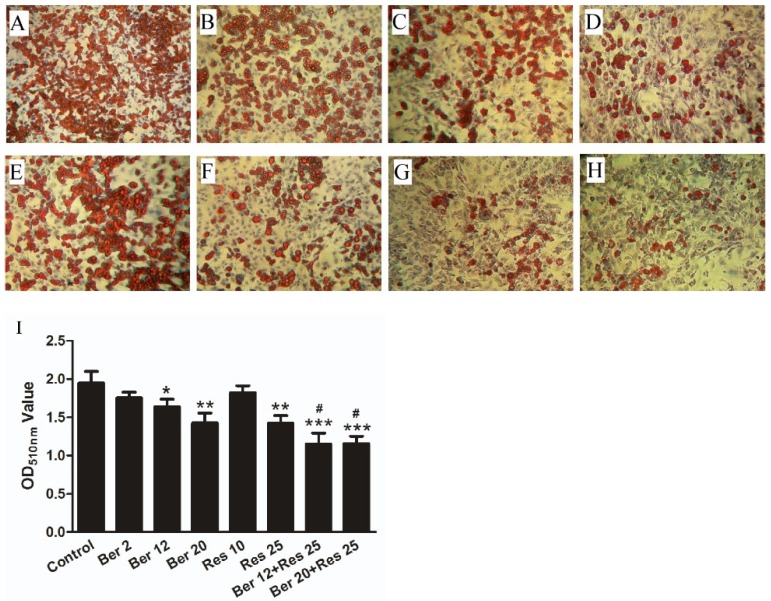
Effect of Ber, Res and their combination on the lipid accumulation in adipocytes. Differentiated 3T3-L1 adipocytes were established by inducing for 10 days (details indicated in Material and Methods) and then treated with different concentration of Ber (**B**–**D**: 2, 12, 20 µmol/L), Res (**E**–**F**: 10, 25 µmol/L) and their combination (**G**–**H**: 12 µmol/L Ber + 25 µmol/L Res, 20 µmol/L Ber + 25 µmol/L Res) for 3 days. Control adipocytes were treated with the vehicle (DMSO < 0.1%) (**A**). Lipid droplets in adipocytes were detected by oil red O staining. The magnification of Microscope for observation was 10 (**I**) the absorbance density of Oil red dye extracted from differentiated adipocytes after Ber and Res treatment. *** *p* < 0.001, ** *p* < 0.01, * *p* < 0.05 vs. control, # *p* < 0.05 vs. single drug. Data were presented as mean ± SD from three separate experiments.

The effects of Ber, Res and their combination on intracellular lipid accumulation were also tested in the established 3T3 L1 adipocytes. As shown in Figure 2, the treatment of Res or Ber alone reduced intracellular lipid accumulation in a dose-dependent manner. However, the lipid accumulation decreased by 40% and 48%, respectively, after treatment with the combination of Res (25 µmol/L) and Ber (12 or 20 µmol/L). The results revealed a more significant lipid-accumulation-reducing effect by the combination therapy than that of any monotherapy (*p* < 0.05).

### 2.2. Resveratrol Increased the Accumulation of Berberine in Hepatic L02 Cells

**Figure 3:**
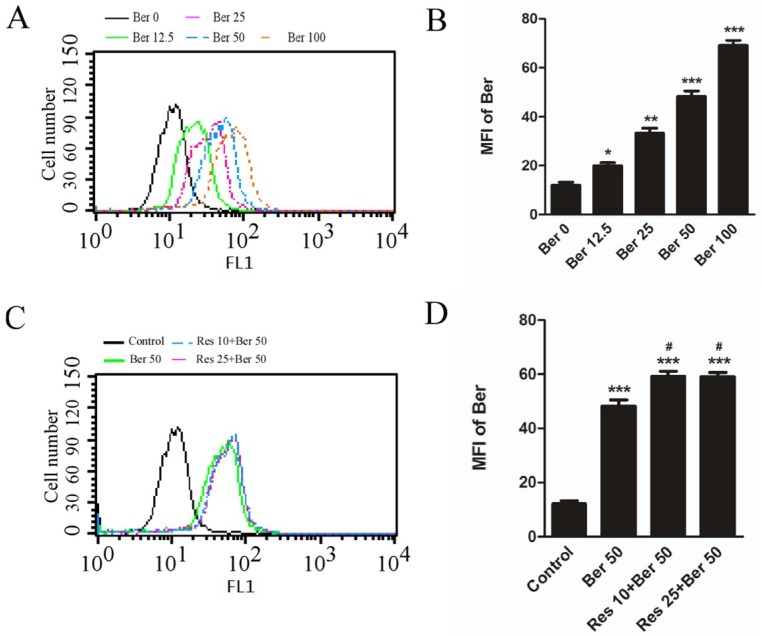
Intracellular accumulation of Berberine enhanced by Res in Hepatic L02 cells. (**A**,**B**) intracellular fluorescence of Ber in a dose-dependent manner (0–100 µmol/L), and mean fluorescence intensity (MFI) calculated and compared in different dose of Ber groups. *** *p* < 0.001, ** *p* < 0.01, * *p* < 0.05 vs. untreated control. (**C**,**D**) Res at 10 or 25 µmol/L added in advance (details indicated in Material and Methods) enhanced intracellular fluorescence of Ber at 50 µmol/L, MFI calculated and compared, *** *p* < 0.001 vs. untreated control, ^#^ *p* < 0.05 vs. single Ber treatment. Data shown are representative of three separate assays.

Ber can be detected by flow cytometry after uptake and accumulation in living cells because of its fluorescence property [15,16]. Indeed, as shown in Figure 3A, the mean fluorescence intensity (MFI) of Ber was increased significantly in a dose-dependent manner. For example, the MFI value of Ber in hepatic cells after treatment with 50 µmol/L was almost four-fold that of control and 2.4 times that of treatment with 12.5 µmol/L Ber. Intriguingly, MFI value in hepatic cells after exposure to 50 µmol/L Ber and 10 or 25 µmol/L Res was increased significantly to 1.4 times that of the single Ber treatment, and the results revealed that resveratrol could increase the accumulation of berberine in hepatocytes.

### 2.3. The Combined Therapy Can Increase Low-Density-Lipoprotein Receptor Expression in Hepatocytes

**Figure 4:**
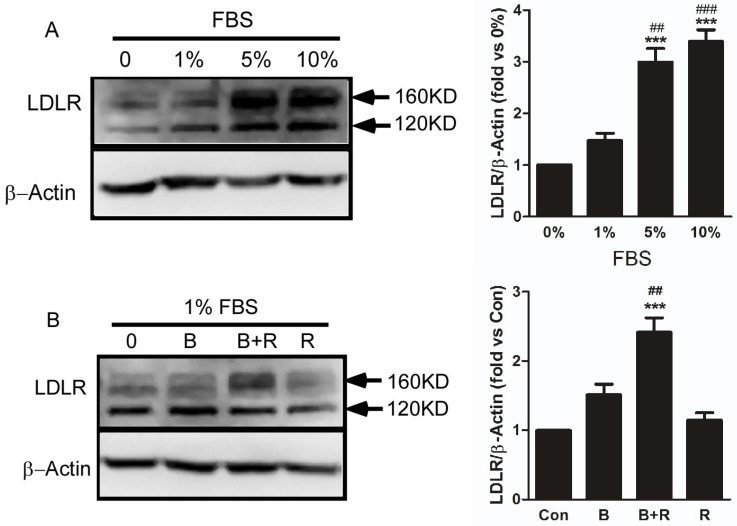
The combination of berberine with resveratrol increased LDLR expression in HepG2 cells. Cells were cultured in 6-well plate for 24 h with a 3 × 10^5^ cell density, and then culture medium were replaced by fresh medium containing different concentration of FBS indicated in A, or 1% FBS and drugs indicated in B for another 24 h followed by extraction of the total proteins from the cells. **A**: the effect of different concentration FBS on LDLR expression were analyzed by western blot assay. Then the band intensity was quantified by grey scanning analysis, and the intensity ratio of LDLR to β-actin in 0% FBS group was set to 1. *** *p* < 0.001 vs. 0% FBS group, ^###^ *p* < 0.001, ^##^ *p* < 0.01 vs. 1% FBS group. **B**: effect of the combination 12 µmol/L berberine (B) with 10 µmol/L resveratrol (R) on LDLR expression. The intensity ratio of LDLR to β-actin in untreated control group was set to 1. *** *p* < 0.001 vs. untreated control, ^##^ *p* < 0.01 vs. single Ber or Res treatment. The results are representative of three separate experiments.

It was reported that the cholesterol lowering effect of Ber was closely associated with its regulation of low-density-lipoprotein receptor (LDLR) expression [6]. Therefore, the effect of combination of Ber with Res on LDLR expression was analyzed. Firstly, the LDLR expression in cells cultured with different concentrations of fetal bovine serum (FBS) was examined in order to evaluate the effects of FBS. As shown in Figure 4A, the expression level of LDLR increased gradually in a serum concentration-dependent manner. When the serum concentration exceeds 5%, the expression of the LDLR receptor no longer increases. Therefore, in order to exclude interference of FBS on LDLR expression, culture medium with 1% FBS was used to detect the effects of the combination of Res and Ber on LDLR expression. The expression of mature LDLR in the combined treatment group (Ber at 12 µmol/L and Res at 10 µmol/L) significantly increased by 50% compared with the single treatment group as revealed by Figure 4B.

## 3. Discussion

Numerous active nature compounds extracted from medicinal plants are used for treatment of dyslipidemia in some diseases, such as morbid obesity or metabolism syndrome, many of which are the sources of currently prescribed synthetic drugs [17]. The present study was to explore the efficacy and possible mechanisms of the combination of nature compounds, Berberine and Resveratrol. Our results demonstrated that the combination of two agents exerted a significant enhanced effect on lipid-lowering in hyperlipidemic mice and intracellular lipid accumulation-decreasing in adipocytes as compared to individual agent alone, which was closely associated with SIRT1 and LDLR. 

Berberine is an alkaloid isolated from many plants, such as *Coptidis Rhizoma*, *Hydrastis Canadensis*. It was found that Ber could be not only a new cholesterol-lowering drug by stabilizing LDLR messenger RNA [6], but also ameliorate whole dysregulation of lipid metabolism to decrease development of fatty liver by activating AMP-dependent protein kinase (AMPK) and regulating SIRT1 in animal experiments [18,19]. Resveratrol, a polyphenol from several plants, has showed beneficial effect on obesity, diabetes and cardiovascular disease as a SIRT1 activator. Reports have also showed that Res and cholesterol-lowering drug statin in combination not only enhanced the lipid-lowering efficacy, but also increase myocardial protection in hypercholesterolemia rats [13,14,20]. Owing to their common target, SIRT1, we speculated that a better efficacy of regulating lipid metabolism may be achieved by Ber and Res in combination. Indeed, in vivo results showed that when combined with Res at 20 mg/kg/day, the dose of Ber reduced by 70% still achieved an effective activity of lowering plasma LDL-c, TG and TC in hyperlipidemia mice, which exhibited significant 27.4% decrease in TC and 31.6% decrease in LDL-c (*p* < 0.05), but no significance in TG. However, monotherapy of Ber at 30 mg/kg/day or Res at 20 mg/kg/day had not significant effect on decrease of plasma LDL-c, TC and TG in hyperlipidemia mice. As compared with monotherapy, combination therapy showed significant 18.9% and 24.1% decrease in TC and LDL-c of, respectively (*p* < 0.05). In animal studies, the dose of hypolipidemic efficacy of Ber was commonly used at 100 mg/kg/day or above [6,21]. However, in our study, the only less one third of 100 mg/kg/day Ber administration in combination with Res could also exhibit a significant lipid-lowering effect. In addition, in differentiated 3T3-L1 adipocytes, our results showed that a decrease of intracellular lipid accumulation was observed in adipocytes treated with Ber or Res alone in a dose dependent manner. However, the lipid accumulation was significantly decreased when adipocytes were treated with Ber at 12 µmol/L or 24 µmol/L combined with Res at 25 µmol/L, which was better than single drug treatment (*p* < 0.05). These data suggested that combination therapy may be a better way to clinic application of hyperlipidemia than monotherapy of Ber because of efficacy at low dose.

Although there are some adverse effects of Ber in clinic monotherapy for hyperlipidemia and diabetes, some studies revealed that multidrug resistance of Ber may occur in a long-term single administration because of Ber being the substrate for p-glycoprotein, which resulted in a decrease of its efficacy in LDLR regulation [9,22,23]. Moreover, in previous studies, it showed that Res had a reversal effect on multidrug resistance of cancer chemotherapy by inhibition of p-glycoprotein function or downregulation of p-glycoprotein expression [24,25]. Therefore, it was interesting to examine whether the enhanced effect of their combination was partially related with the increased accumulation of Ber in cells caused by Res. As a fluorescent dye for cell, mitochondria and DNA analysis, Ber can be detected through flow cytometry at 550 nm under excitation light [15,16]. As shown in Figure 3, the mean fluorescence intensity (MFI) of hepatocytes after treatment with Ber increased in a concentration-dependent manner, which reflected the accumulation of intracellular Ber. After exposure to the combined agents, the MFI values of cells increased more significantly than that of the single Ber treatment. Meanwhile, in some reports [26] or our study, single Res showed a non-fluorescent property in hepatocytes detected by flow cytometry (Figure S2). Therefore, the increased MFI values were due to the increased accumulation of Ber within the cells. However, the intracellular accumulation of Ber remained unchanged when the dose of resveratrol increased. This data implied that the enhanced effect of the combination may be partially explained by the increased accumulation of Ber in cells that was caused by Res treatment.

One of the important mechanisms for hypolipidemic function of Ber is the involvement in cholesterol-lowering by upregulation LDLR expression [6]. The formation process of functional LDLR begins from a precursor-like receptor in endoplasmic reticulum to a mature receptor after being *O*-linked glycosylated in the Golgi apparatus, and migrating to cell membrane [2]. Meanwhile, it was reported that serum factors could upregulate LDLR expression [27]. This was confirmed by our study that normal concentration of serum (10%) in cell culture medium have a direct effect on increasing the level of mature LDLR. To remove the influence of serum, the cells were cultured in medium with low concentration of serum (1%), and then were treated with Ber plus Res. The results revealed that the combination treatment could significantly increase the level of mature LDLR compared with single Ber treatment. These data implied that the reason for enhanced efficacy of combination may be associated with increase of the intracellular accumulation of Ber by Res, which enhanced hypolipidemic effect by Ber-induced LDLR expression. However, in some report, deletion of SIRT1 could decrease the level of LDLR mRNA and a SIRT1-specific activator could enhance LDLR expression [28,29]. In view of the upregulation SIRT1 protein of Ber (Figure S1) and activation SIRT1 enzyme activity of Res, another explanation may be not rule out that SIRT1 targeted by Ber and Res may be partially participating in upregulation of LDLR expression.

In conclusion, our results demonstrated that berberine at low dose in combination with resveratrol may be a better therapy than monotherapy during the lipid metabolism disorder scenario, which was closely associated with enhanced LDLR expression by Res-mediated intracellular accumulation of Ber. It might imply that this combination could be an effective trial for clinical obesity-associated disease therapy, such as type II diabetes, metabolic syndrome.

## 4. Materials and Methods

### 4.1. Experimental Animals and Study Protocol

Female C57BL/6J mice were purchased from Beijing Vital River Laboratory Animal Technology Co., Ltd (Beijing, China) at 7–8 weeks of age, accommodated for one week after receipt, and maintained at a temperature and humidity of 25 °C and 55%, respectively and a 12 h:12 h light:dark cycle. All mice were randomly assigned to either control low fat diet (LFD) (Research Diets number D12450J, 10% kcal from fat) or high fat diet (HFD) (Research Diets number 12,492, 60% kcal from fat) for 12 weeks. Then Mice with HFD were divided randomly into five groups and administrated orally with different dose of drugs for 4 weeks: (1) 0.9% saline at equal volume, (2) Ber at 30 mg/kg/day, (3) Res at 20 mg/kg/day, (4) Ber at 100 mg/kg/day, (5) Ber at 30 mg/kg/day plus Res at 20 mg/kg/day. Ber was administrated twice a day at 8 A.M. and 5 P.M., respectively, whereas Res was given once a day at 5 P.M. Berberine and Resveratrol was purchased from Hangzhou Huadong Medicine Group Kangrun Pharmaceutical Co., Ltd. (Hangzhou, China).

Blood samples were taken after the treatment by intraocular canthus venous plexus after 6-h fast. Serums were isolated by centrifugation. Serum LDL-c, total cholesterol (TC), and triglyceride (TG) levels were assayed by Standard enzymatic methods with commercially available kits purchased from Najing jiancheng Bioengineering institute (Nanjing, China). All animal procedures and experiments were conducted in accordance with Chinese Administration Rule of Laboratory Animal and approved by the Ethics Committees and the Research Committees of Xinxiang Medical University (81373135-2014, 15 March 2014).

### 4.2. Cell Culture and Adipocytes Differentiation

Human hepatic L02 and HepG2 cells were cultured in RPMI-1640 medium containing 10% fetal bovine serum, 100 IU/mL penicillin, and 100 µg/mL streptomycin. 3T3-L1 preadipocytes were cultured in Dulbecco’s Modified Eagle Medium (DMEM) containing 15% fetal bovine serum, 100 IU/mL penicillin, and 100 µg/mL streptomycin. All cells were cultured in an incubator with a humidified atmosphere of 5% CO2, 95% air at 37 °C. The 3T3-L1 differentiation was performed as previously described [30]. Briefly, 3 × 10^4^ 3T3-L1 cells were plated in 60 mm dishes, grown to confluence and fed every two days during growth. At two days of post-confluence (day 0), the medium supplemented with methyllisobutylxanthine (0.5 mM), dexamethasone (1 µM), and insulin (10 g/mL) was added to dishes for 2 days induction (day 2). In addition, then the medium was replaced with a new medium supplemented only with insulin (10 g/mL) for another 2 days. At day 4, the medium was changed to complete DMEM medium for maintenance. At day 10, differentiated 3T3-L1 adipocytes were treated with Ber, Res and their combination for 3 days. Berberine and Resveratrol for cell assay was purchased from Selleck Chemicals company (Shanghai, China).

### 4.3. Oil Red O Staining Assay

The intracellular lipid accumulation of differentiated cells was detected by oil red O staining. Cells were washed with phosphate buffered saline (PBS) twice, fixed in 10% formalin for 15 min at room temperature. After washed with PBS, cells were stained with oil red O for 20 min, then rinsed with PBS twice and 60% isopropanol once. Cell nuclei were stained with hematoxylin. After dyed, cells were washed and overlaid with PBS, photographed using a phase contrast microscopy. For semi-quantitative analysis of oil red O staining, the dye in intracellular lipid droplets was extracted by incubating cells stained with 60% isopropanol containing 4% Sodium dodecyl sulfate (SDS) at room temperature for 20 min. The optical density of oil red O were monitored spectrophotometrically at 510 nm wavelength.

### 4.4. Western Blot Assay

After treatment, cells pellets collected immediately were lysed in RIPA cell lysis buffer (10 mM Tris-HCl pH 7.4, 1% Triton X-100, 1% sodium deoxycholate, 0.1% SDS, 160 mM NaCl, 5 mM EDTA, 50 mM NaF, 10% Glycerol, 1 mM Na_3_VO_4_, and 5 mM sodium pyrophoshate). The total proteins extracted from cells were quantified by bicinchoninic acid (BCA) protein assay. Equal amounts of protein samples were subjected to 8–10% sodium dodecyl sulfate–polyacrylamide gel electrophoresis (SDS-PAGE) and transferred onto Polyvinylidene fluoride (PVDF) membrane. The membranes were incubated with various antibodies at 1:1000 dilutions. Then they were incubated with horseradish peroxidase-conjugated secondary antibodies at 1:2000 dilutions. Visualization was detected with ECL Plus Western Blotting Detection System according to the manufacturer’s recommendation. Goat antibodies directed against LDLR (C-7, sc-18823), HRP-labeled anti-goat IgG (sc-2354) and anti-β-actin antibody (sc-8432) were from Santa Cruz Biotechnology (Heidelberg, German). SIRT1 Antibody (#2493) were from Cell Signal Technology (Shanghai, China).

### 4.5. Flow Cytometry Assay

L02 cells were seeded into six-well plates at a density of 4 × 10^5^ /well for 24 h culture. For the intracellular fluorescence of Ber assay, a range of Ber concentrations (12.5–100 µmol/L) was added to dishes of L02 cells for 2 h, and then cells were measured with a FACScan flow cytometer (Shanghai, China) using the FL1 filter set after trypsinization, washing and resuspension in PBS with 5% fetal bovine serum. For combination studies, L02 cells were pretreated with 10 or 25 µmol/L Res for 2 h, and then co-treated with 50 µmol/L Ber for 2 h. The intracellular fluorescence of Ber was detected by flow cytometry.

### 4.6. Statistical Analysis.

Data analysis was performed using GraphPad Prism 5 (GraphPad software Inc., San Diego, CA, USA) and expressed as mean ± SD. The significance of differences between two groups was determined with student’s-t-test. For multiple comparison, One-way analysis of variance (ANOVA) was used with Turkey’s test. A *p*-value less than 0.05 was considered to be significant.

## Supplementary Materials

Supplementary materials can be found at http://www.mdpi.com/1422-0067/19/12/3903/s1.

## Abbreviations

Ber	Berberine
Res	Resveratrol
SIRT1	Sirtuin 1
TC	Total cholesterol
LDLR	Low Density Lipoprotein Receptor
HFD	High fat diet
TG	triglyceride
LDL-c	Low Density Lipoprotein cholesterol
MFI	mean fluorescence intensity
FBS	Fetal Bovine Serum
DMSO	Dimethyl Sulphoxide
PBS	phosphate buffered saline
SDS-PAGE	Sodium Dodecyl Sulfate–Polyacrylamide Gel Electrophoresis
PVDF	Polyvinylidene fluoride
