# Supplementary material for: Combination of Berberine with Resveratrol Improves the Lipid-Lowering Efficacy
Source: Int J Mol Sci. 2018 Dec 6;19(12):3903. doi: 10.3390/ijms19123903 (PMC6321535; doi:10.3390/ijms19123903)
Supplement: Supplementary file 1 [file ijms-19-03903-s001.pdf]

Supplementary File

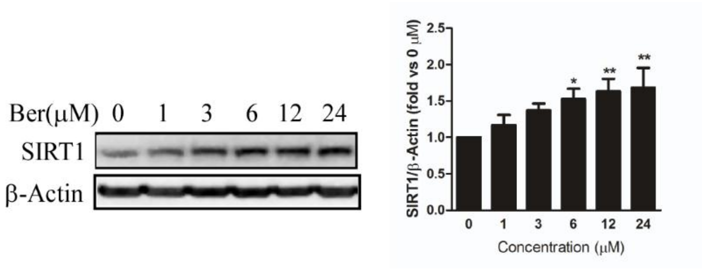

Figure S1. The berberine increased SIRT1 expression in hepatic L02 cells. Cells were cultured in 6-well plate for 24h with a 3×10^5 cell density, and then culture medium were replaced by fresh medium containing different concentrations of Ber indicated for another 24h followed by extraction of the total proteins from the cells. SIRT1 expression were analyzed by western blot assay. The band intensity was quantified by grey scanning analysis, and the intensity ratio of SIRT1 to β-actin in control group was set to 1. $^{**}p<0.01$, $^{*}p<0.05$ vs Ber 0μM group. The results are representative of three separate experiments.

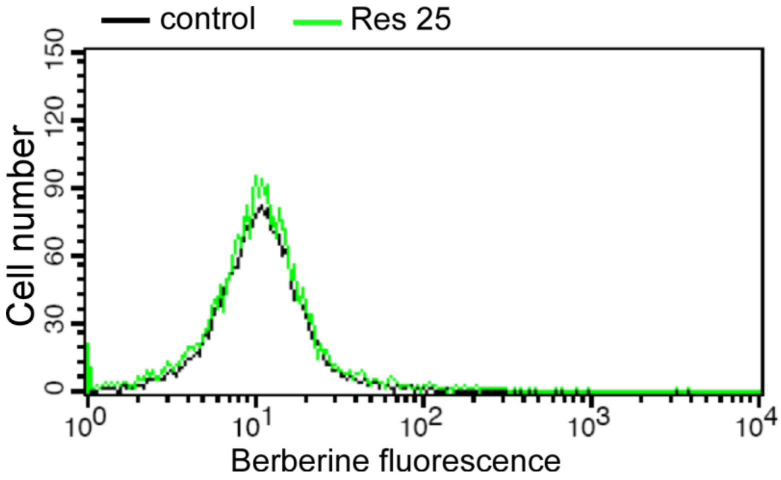

Figure S2. No fluorescence of Res were detected in detection wavelength of Ber. Res at 25μmol/L was added in hepatic L02 cells and intracellular Ber fluorescence was detected by flow cytometry(details indicated in *Material and Methods*). Data shown are representative of three separate assays.
